# Crash Performance of Inward-Inverting Composite Tubes Filled with Foam: Experimentation and Simulation

**DOI:** 10.3390/ma16196378

**Published:** 2023-09-24

**Authors:** Pu Yu, Zhefeng Yu, Xiang Zhou, Wu Xu

**Affiliations:** 1 Shanghai Aircraft Manufacturing Co., Ltd., Shanghai 201324, China; yupu@comac.cc; 2 Aerospace Structure Research Center, School of Aeronautics and Astronautics, Shanghai Jiao Tong University, Shanghai 200240, China; xiangzhou@sjtu.edu.cn (X.Z.); xuwu@sjtu.edu.cn (W.X.)

**Keywords:** shock absorber, inward-inverting composite tube, foam-filled, specific energy absorption

## Abstract

This study presents a novel shock absorber with an inward-inverting composite foam-filled tube. Under the compression of a pressing cap and the action of an internal inversion cap, the composite tube inverted inward. During the crushing, the fronds of the composite tube compacted the foam, thereby enhancing the energy absorption. Three types of foams were applied to the absorber, and a drop-weight impact test was performed to obtain the assessment parameters. The foam increased the specific energy absorption (SEA) of the composite tube to 103 kJ/kg. Finite-element simulation based on the user-defined material subroutine was performed for the initial failure and stable stages of the crushing, and a foam model was identified through the experimental data. The mean crush force from the simulation agreed with the experimental data, and the SEA maximum error was <7%, thus validating the crush simulation of the proposed shock absorber. The development of the damage modes of the plies was analyzed based on the simulation results, showing a good energy absorption mechanism of this shock absorber.

## 1. Introduction

Crashworthiness has become a basic requirement for transportation vehicles, such as helicopters, airplanes, and automobiles [1,2,3]. For structures made of aluminum and other metals, the plastic deformation of the structural members absorbs the kinetic energy transferred during the impact. Composite materials have been widely used to fabricate lighter structures; some of them, such as carbon-fiber-reinforced plastic (CFRP), are brittle; thus, their crashworthiness is a key issue. The kinetic energy of the CFRP structure dissipates through material failures, including fiber breakage, crushing of the matrix, and delamination. For automobiles, crush boxes are typically employed [4], whereas for aircraft, struts in the fuselage serve as energy absorbers [5,6,7]. Composite tubes are widely used in structures such as struts [8,9,10] and bumpers. Some composite tubes are subjected to transverse loads [11,12], whereas other loads are axial. The energy absorption of the former is lower; therefore, most research has focused on the axial crush problem.

The techniques used to trigger stable crushing and enhance the specific energy absorption (SEA) present two key issues. The triggering mechanism at the end of a tube that initiates axial crushing may be “a chamfer” or “steeple” [13,14]. The fiber layout and triggering technique of composite tubes have been studied [15,16,17]. The SEA of CFRP tubes generally ranges from 50 to 80 kJ/kg [16,18,19].

The failure-triggering method can be employed for a composite tube to achieve a higher SEA and a more-stable crushing procedure. A rigid die is typically used to trigger the failure of the tube [20]. Heimbs et al. [21] presented a method wherein a composite column was cut into strips by a special machine under an axial load. Similarly, Ueda et al. [22] proposed a trigger with a double-sided plug, and Tong et al. [23] proposed a chamfer trigger, which guides an external flap of the composite tube. In the abovementioned studies, the crushed debris was spread out across the tube. By contrast, Siromani et al. [24] studied the performance of CFRP tubes under the action of an inward-folding cap under quasi-static loading. Yu et al. [25,26] proposed a shock absorber based on inward-folding composite laminate tubes and sandwich tubes [27]. The debris filled the hollow cavity and further increased the SEA or passed through the hole at the other end. The SEA during the steady-state procedure in the impact test exceeded 80 kJ/kg. The SEA could be further improved by filling the hollow with other substances.

Foam is lightweight; consequently, it is used in some structures to increase energy absorption. Foam can be made from polymers or aluminum. In one study, it was applied to an internally stiffened tube subject to a transverse load [28], where the SEA was below 8 kJ/kg. Yang et al. applied aluminum foam to a skeleton-filled tube subjected to an axial load [29] and achieved an SEA of 72 kJ/kg, which was higher than that when no foam was used (approximately 63 kJ/kg). Alia and Zhou et al. studied a structure consisting of foam reinforced by small carbon fiber tubes; the SEA values in the quasi-static test were close to 93 kJ/kg [30,31]. Therefore, a foam-filled tube is a prospective energy absorber.

Finite-element (FE) analysis of the crushing of composite laminates is also a key issue with many challenges. Although laminates have a complex structure, their failure during crushing can be simulated with shell elements. Feraboli et al. [6] simulated the crushing of a composite specimen with LS-DYNA (R6.0), where the “Enhanced Composite Damage” material model MAT54 was applied to the shell elements, which represented the entirety of the laminates. This model requires experimentally determined parameters, rather than those based on the damage mechanics. Siromani et al. [32] also simulated the crushing of a composite tube using the material model MAT54, where one layer of the shell elements represented several plies. Inward-folding crushing was also simulated. A more-popular method is to establish a model with a stacked shell or brick element, and a cohesive element is typically used to connect different layers to simulate interlaminar damage, even when simulating the crushing procedure of a complex strut in a fuselage [33]. The detailed intralaminar damage, including matrix cracks and fiber failures, can be predicted using the 3D Hashin criteria, which are generally applied to the material through the coded user-defined material subroutine (VUMAT) in ABAQUS/Explicit [34]. The interlaminar damage (primarily delamination) is simulated using cohesive zone elements [35,36,37].

This study proposes a shock absorber with an internal inversion cap [25,26,27], for which the SEA exceeded 80 kJ/kg under dynamic testing. Herein, the tube was filled with polymer foam to reinforce it. During crushing, the folded fronds of the composite tube in also compacted the foam, thereby enhancing the energy absorption. The remainder of this paper is organized as follows. Section 2 presents the shock absorber with the three types of foam used and the experimental method employed. Section 3 describes the FE simulation of specimen crushing. In Section 4, the results of the experiment and simulation are given and discussed. Finally, Section 5 provides the conclusions of the study.

## 2. Experimental Setup

### 2.1. Configuration of the Energy Absorber

**Figure 1:**
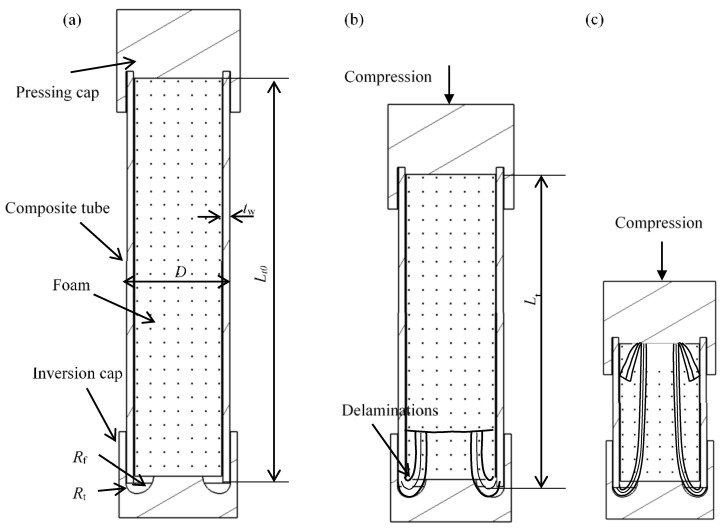
Concept of the shock absorber: (**a**) configuration of the structure, (**b**) stable crushing stage, and (**c**) the compression of composite debris after it reaches the pressing cap.

**Figure 2:**
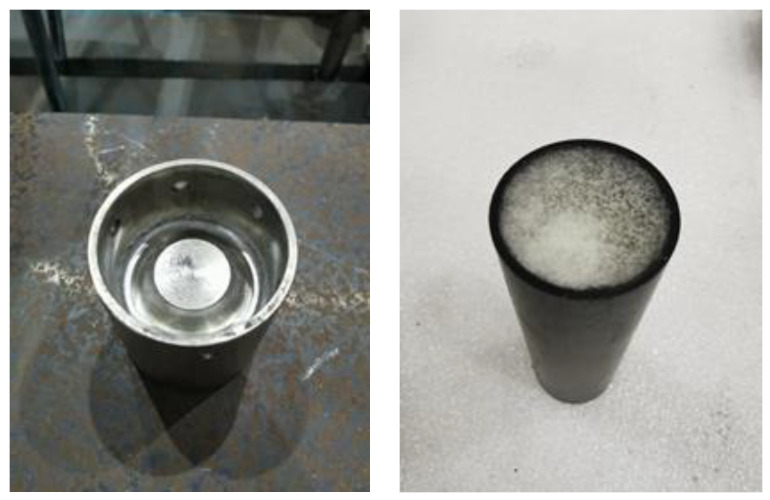
Inversion cap (**left**) and foam-filled composite tube (**right**).

A schematic of the energy absorber is shown in Figure 1a. It consisted of a composite tube filled with polymer foam, an internal inversion cap, and a pressing cap. A composite tube with a length of $L_{t0}$, a diameter of *D*, and a thickness of $t_{w}$ was connected to the two caps. The internal inversion cap destructed the composite tube through a triggering fillet with a radius $R_{t}$ and folded the destructed tube wall through the inverted surface with a radius $R_{f}$. Herein, the inverted composite fronds continued to compress the foam (Figure 1b). When the folded tube reached the internal inversion cap (Figure 1c), composite debris began filling the cavity, thereby increasing the reaction load. The metal inversion cap and composite tube filled with foam are shown in Figure 2.

### 2.2. Parameters of Shock Absorber

The behavior of a shock absorber without foam reinforcement was demonstrated previously in [25,26], where the internal inversion cap with $R_{t}$ = 3 mm and $R_{f}$ = 5 mm provided a steady crush triggering and high SEA; therefore, the same internal inversion cap was adopted in this study. The composite tube was mainly made of carbon fiber/epoxy prepreg unidirectional tape, and a layer of plain woven fabric was coated on the outside of the tube, which was not considered in the numerical simulation in this study. Two thicknesses of 2 and 1.5 mm with ply setups of [90/0]_5_ and [(0/90)_3_0] were considered, where the tape at 0° coincides with the axis of the tube. The composite tubes had a length of 100 mm and a diameter *D* of 30 mm. The other mechanical parameters of the unidirectional tape are listed in Table 1.

**Figure 3:**
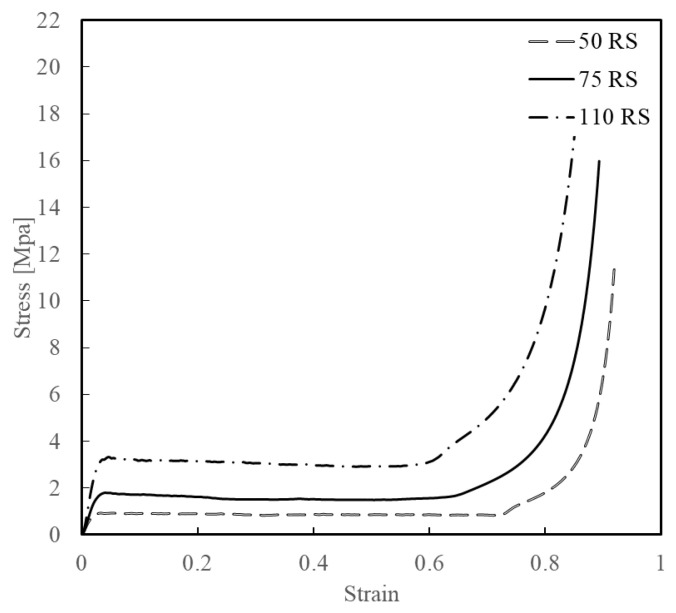
Stress–strain curves of PMI foam samples.

The mechanical properties of the three types of polymethacrylimide (PMI) foam provided by the supplier are listed in Table 2. The parameters needed for the FE simulation through ABAQUS were calculated by the embedded code based on the input stress–strain data, whereas the stress–strain curves were obtained from static tests conducted on the MTS servo-hydraulic system, as shown in Figure 3.

Eight types of absorbers were fabricated by combining the abovementioned composite tube and foam, as listed in Table 3. Two or more samples of each type were prepared and tested.

### 2.3. Equipment for Impact Tests

**Figure 4:**
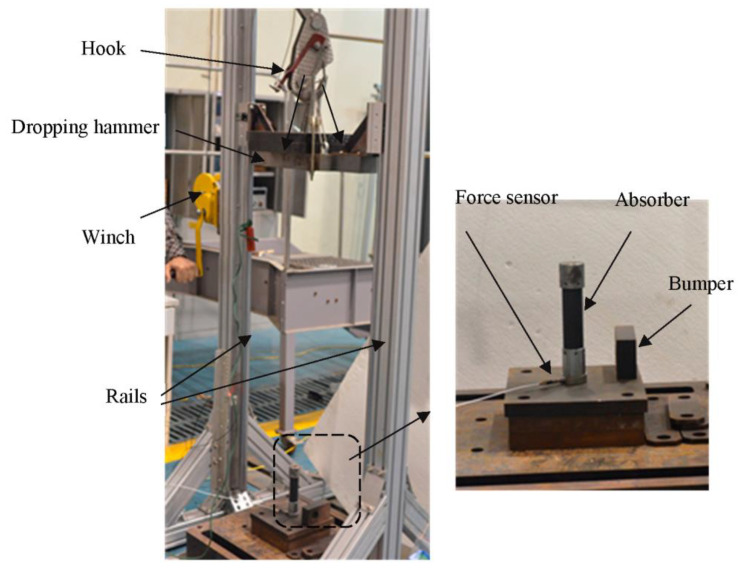
Setup of drop-weight tower for impact test.

Impact tests on the shock absorbers were conducted using a drop-weight tower, as introduced in [26]; see Figure 4. The weight of the dropping hammer ranged from 30 to 90 kg, and the lift height ranged from 1 to 1.5 m, depending on the energy necessary to test the different absorbers. To evaluate the energy absorbed, crush distance and other characteristics of the absorber, the impact velocity $v_{i}$ was measured using an optical sensor; the impact load *F*(*t*) was measured using a dynamic force sensor, a PCB 200C20 mounted at the bottom of absorber.

A DH-5922 digital data acquisition system was used to record the impact force F(t) and the impact velocity at a sampling rate of 100 kHz. The velocity of dropping hammer at time t  was obtained by [38]

(1) $$v(t)=v_{i}+gt-\int_{0}^{t}\frac{F(t)}{m}dt,$$

where *g* denotes the gravitational acceleration; m denotes the hammer mass. The crushing length at time t is expressed by:

(2) $$\delta_{t}=v_{t}+\frac{gt^{2}}{2}-\int_{0}^{t}\left(\int_{0}^{t}\frac{F(t)}{m}dt\right)dt.$$

In order to compare the behavior of the absorbers with different lengths, the dimensionless distance is defined as:

(3) $$\bar{\delta_{t}}=\frac{\delta_{t}}{L_{t0}}=\frac{L_{t0}-L_{t}}{L_{t0}},$$

where $L_{t0}$ and $L_{t}$ are the original and real-time tube length, respectively, as shown in Figure 1.

Based on the impact load and crushing length, several criteria used to assess the energy absorption abilities of thin-walled columns can be calculated. The SEA is the value of the energy absorbed per unit mass; for a composite tube, it can be expressed as:

(4) $$\mathrm{SEA}=\frac{\int_{0}^{\delta_{C}}F(\delta )d\delta }{\bar{m}\delta_{C}},$$

where $\delta_{C}$ denotes the concerned crushing length; $\bar{m}$ denotes the mass per unit length; and $F\left(\delta \right)$ is the load at crush distance δ. The mean crush force (MCF) over the crush distance is given as:

(5) $$\mathrm{MCF}=\frac{1}{\delta_{C}}\int_{0}^{\delta_{C}}F(\delta )d\delta .$$

The crushing force efficiency (CFE) is the ratio of the MCF to the maximum initial collapse force (MICF).

(6) $$\mathrm{CFE}=\frac{\mathrm{MCF}}{\mathrm{MICF}}.$$

The following load should not be higher after the MICF. A high CFE implies that the energy absorber can absorb more energy with a relatively lower reaction load to the occupants, which further suggests that the material efficiency is high.

## 3. Numerical Simulation

### 3.1. Model of Intralaminar Damage in Composite Tube

The plies of the composite tube were meshed using eight-node C3D8R reduced integration. The intralaminar damage was simulated using the ABAQUS/Explicit solver through a user material VUMAT subroutine developed in the FORTRAN language. Failure initiation was determined based on the Chang–Lessard failure criteria [39,40], which include four failure modes: matrix crushing, matrix cracking, fiber–matrix shearing failure, and fiber failure. In this study, fiber compressive failure was also considered. The failure criteria are listed in Table 4, and their strength parameters are listed in Table 1.

**Figure 5:**
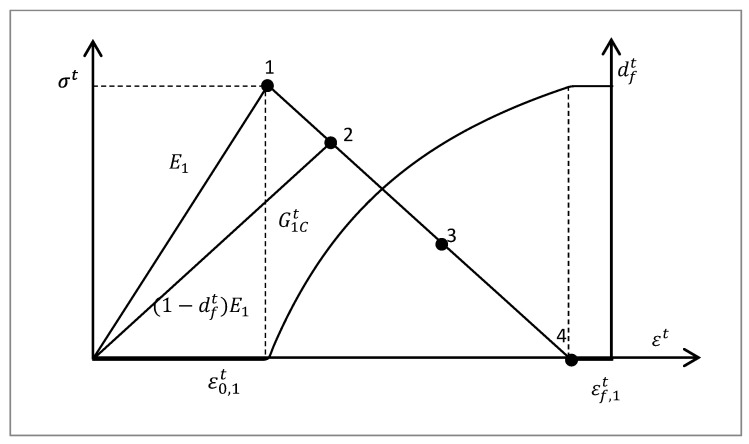
Intralaminar damage behavior model for tensile failure mode.

Herein, a linear stress–strain behavior was assumed for the composite laminar damage, demonstrated with the tensile stress shown as Figure 5. Once the damage was initiated, the stress began to reduce to zero linearly as the tensile strain increased. The failure initiation strain in tension $\varepsilon_{0,1}^{t}$ is

(7) $$\varepsilon_{0,1}^{t}=\frac{X_{T}}{E_{1}}.$$

The maximum strain $\varepsilon_{f,1}^{t}$ is given as

(8) $$\varepsilon_{f,1}^{t}=\frac{2G_{1C}^{t}}{\sigma_{t}l},$$

where $G_{1C}^{t}$ is the fracture toughness, which equals the area below the strain–stress curve, including the linear and failure procedure; $\sigma^{t}$ is the ultimate tensile strength of the material; and l is the characteristic length of the crack growth, which is relative to the element volume, as the fracture energy is distributed over the volume of the represented element. Herein, in the VUMAT subroutine, the cube root of the element volume was transferred through the variable *charLength*. The element had the same width and length, and the thickness t was 0.2 mm; thus, $l=\sqrt{{charLength}_{}^{3}/t}$.

The damage variable in the tension of the fiber also includes the effect of shear strain, which is expressed as follows.

(9) $$d_{f}^{t}=\frac{\varepsilon_{f,1}^{t}}{\varepsilon_{f,1}^{t}-\varepsilon_{0,1}^{t}}\left(1-\frac{1}{r_{ft}}\right),$$

where

(10) $$r_{\mathrm{ft}}=\sqrt{{\left(\frac{S_{11}}{\varepsilon_{0,1}^{t}E_{1}\left(1-d_{f,\mathrm{old}}^{t}\right)}\right)}^{2}+{\left(\frac{\varepsilon_{12}}{\varepsilon_{12}^{c}}\right)}^{2}+{\left(\frac{\varepsilon_{13}}{\varepsilon_{13}^{c}}\right)}^{2}},$$

where $d_{f,old}^{t}$ is the damage coefficient of the last step; and $\varepsilon_{12}^{c}\;and\;\varepsilon_{13}^{c}$ are the strains at damage initiation. The same damage evolution was applied to the damage of the unidirectional tape in 2 and 3 directions. Then, the material stiffness matrix could be obtained as follows.

(11) $$C_{d}=\left[\begin{array}{cccccc} \alpha C_{11} & \alpha \beta C_{12} & \alpha \psi C_{13} & 0 & 0 & 0\\ \alpha \beta C_{12} & \beta C_{22} & \beta \psi C_{23} & 0 & 0 & 0\\ \alpha \psi C_{13} & \beta \psi C_{23} & \psi C_{33} & 0 & 0 & 0\\ 0 & 0 & 0 & \alpha \beta C_{44} & 0 & 0\\ 0 & 0 & 0 & 0 & \alpha \psi C_{55} & 0\\ 0 & 0 & 0 & 0 & 0 & \beta \psi C_{66} \end{array}\right],$$

where $\alpha =(1-d_{f}^{t})(1-d_{f}^{c})$; $\beta =(1-d_{m}^{t})(1-d_{m}^{c})$; $\psi =(1-d_{d}^{t})(1-d_{d}^{c})$; superscripts t and c denote the tension and compression, respectively; subscripts f, m, and d denote the 1, 2, and 3 directions of the unidirectional tape. In fact, the maximum of every damage variable was limited to approximately 0.8 to avoid the distortion of elements and maintain enough stiffness for simulating the supporting effect of composite debris. The element was removed when the strain was greater than 0.8 or less than −0.6, which was calculated using the tensor matrix transferred by variable stretchNew in the VUMAT subroutine.

### 3.2. Model of Interlaminar Damage in Composite Tube

**Figure 6:**
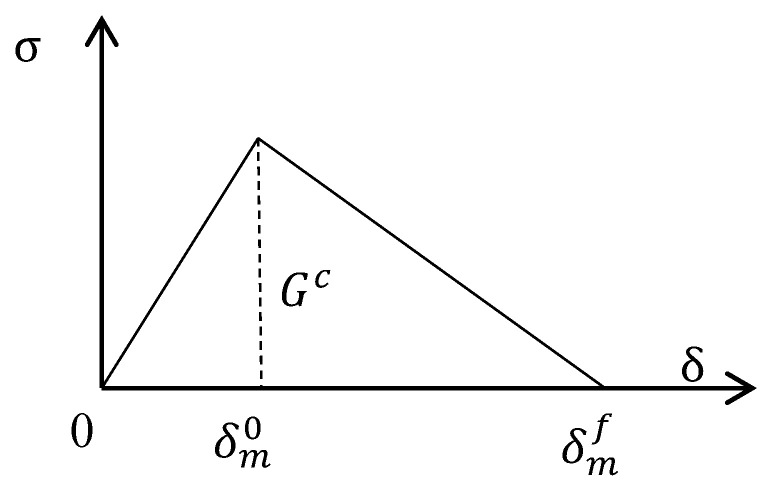
Law of the traction–separation for the cohesive zone.

To simulate the separation of the plies, the cohesive elements (COH3D8) were established between adjacent layers to simulate their interaction and the interlaminar damage. The delamination onset was determined by the traction–separation law shown in Figure 6, where $\delta_{m}$ is the equivalent displacement, given by [41]

(12) $$\delta_{m}=\sqrt{{\langle \delta_{n}\rangle }^{2}+\delta_{s}^{2}+\delta_{t}^{2}},$$

where $\delta_{n}$ is the normal displacement, with $\langle \delta_{n}\rangle =(\delta_{n}+\left|\delta_{n}\right|)/2$; $\delta_{s}$ and $\delta_{t}$ are the in-plane shear displacements. The cohesive stresses $t_{n}$, $t_{s}$, and $t_{t}$ were obtained as follows.

(13) $$\{\begin{array}{c} t_{n}\\ t_{s}\\ t_{t} \end{array}\}=\frac{1}{T_{0}}\left[\begin{array}{ccc} E_{\mathrm{nn}} & & \\ & E_{\mathrm{ss}} & \\ & & E_{\mathrm{tt}} \end{array}\right]\{\begin{array}{c} \delta_{n}\\ \delta_{s}\\ \delta_{t} \end{array}\},$$

where $E_{ii}$(*i* = n, s, t) is the elasticity coefficient, as listed in Table 5, and $T_{0}$ is the thickness of the cohesive element, which was set as 0.01 mm in this study.

The crack occurred when the quadratic interaction criterion on stress was satisfied:

(14) $${\{\frac{\langle t_{n}\rangle }{t_{n}^{0}}\}}^{2}+{\left\{\frac{t_{s}}{t_{s}^{0}}\right\}}^{2}+{\left\{\frac{t_{t}}{t_{t}^{0}}\right\}}^{2}\geq 1;\;\langle t_{n}\rangle =\frac{t_{n}+\left|t_{n}\right|}{2},$$

where $t_{n}^{0}$, $t_{s}^{0},\;{and\;t}_{t}^{0}$ denote the peak values of the nominal direct, first shear, and second shear stress on the interface, respectively.

The Benzeggagh–Kenane formulation based on the fracture energy [42] was used to describe the damage evolution, as follows.

(15) $$G^{C}=G_{n}^{C}+\left(G_{s}^{C}-G_{n}^{C}\right){\left(\frac{G_{s}+G_{t}}{G_{n}+G_{s}+G_{t}}\right)}^{\eta },$$

where $G_{n}^{C}$, $G_{s}^{C}$, and $G_{t}^{C}$ are the values of the critical fracture energy per unit area necessary to initiate failure with the normal fracture modes and the two in-plane modes; $G_{n}$, $G_{s}$, and $G_{t}$ are the values of fracture energy; and η ranges from 0.5 to 2.0 and was determined to be 2.0 in this study.

Linear damage softening behavior was adopted; the damage evolution variable is expressed as:

(16) $$D=\frac{\delta_{m}^{f}\left(\delta_{m}^{\max}-\delta_{m}^{0}\right)}{\delta_{m}^{\max}\left(\delta_{m}^{f}-\delta_{m}^{0}\right)},$$

where $\delta_{m}^{0}$ is the effective displacement at damage initiation; $\delta_{m}^{max}$ is the maximum value of the effective displacement in the loading procedure; and $\delta_{m}^{f}$ is the effective displacement at complete failure, with $\delta_{m}^{f}=2G^{C}/T_{eff}^{0}$, where $T_{eff}^{0}$ is the effective stress at damage initiation. Once the overall damage variable reached the specified value (0.85 in this study), the element was removed from the model. The stress components are affected by damage as follows.

(17) $$\begin{array}{c} t_{n}=\{\begin{array}{c} \left(1-D\right){\bar{t}}_{n},{\bar{t}}_{n}\geq 0\\ {\bar{t}}_{n},\mathrm{otherwise} \end{array}\\ t_{s}=\left(1-D\right){\bar{t}}_{s}\\ t_{t}=\left(1-D\right){\bar{t}}_{t} \end{array},$$

where ${\bar{t}}_{n}$, ${\bar{t}}_{s}$, and ${\bar{t}}_{t}$ are the stress components calculated according to the traction–separation law for the current strains without damage.

### 3.3. Model of Foam Behaviors

A hyperfoam material was used to simulate the behavior of the PMI foam. The elastic behavior of the foam is based on the strain energy function:

(18) $$U=\sum_{i=1}^{N}\frac{2\mu_{i}}{\alpha_{i}^{2}}\left[{\hat{\lambda }}_{1}^{\alpha_{i}}+{\hat{\lambda }}_{2}^{\alpha_{i}}+{\hat{\lambda }}_{3}^{\alpha_{i}}-3+\frac{1}{\beta_{i}}\left({\left(J^{\mathrm{el}}\right)}^{-\alpha_{i}\beta_{i}}-1\right)\right],$$

where *N* is a material parameter less than 6; and $\mu_{i}$, $\alpha_{i}$, and $\beta_{i}$ are temperature-dependent material parameters.

(19) $${\hat{\lambda }}_{i}={\left(J^{\mathrm{th}}\right)}^{-\frac{1}{3}}\lambda_{i},$$

where $\lambda_{i}$ is the principal stretches and $J^{el}$ is the elastic volume ratio, expressed as follows.

(20) $$J^{\mathrm{el}}=\frac{J}{J^{\mathrm{th}}}={\hat{\lambda }}_{1}{\hat{\lambda }}_{2}{\hat{\lambda }}_{3},$$

where *J* is the volume ratio of the current volume to the reference volume and $J^{th}$ is the thermal volume ratio, expressed as follows.

(21) $$J^{\mathrm{th}}={\left(1+\varepsilon^{\mathrm{th}}\right)}^{3},$$

For each term in the energy function, the coefficient $\beta_{i}$ determines the degree of compressibility, which is related to Poisson’s ratio, $\nu_{i}$, as follows.

(22) $$\beta_{i}=\frac{\nu_{i}}{1-2\nu_{i}},$$

(23) $$\nu_{i}=\frac{\beta_{i}}{1+2\beta_{i}}.$$

In this study, *N* was determined to be three, and the abovementioned material parameters were calculated in ABAQUS when the test data shown in Table 6 were specified.

## 4. Results and Discussion

### 4.1. Experimental Results

**Figure 7:**
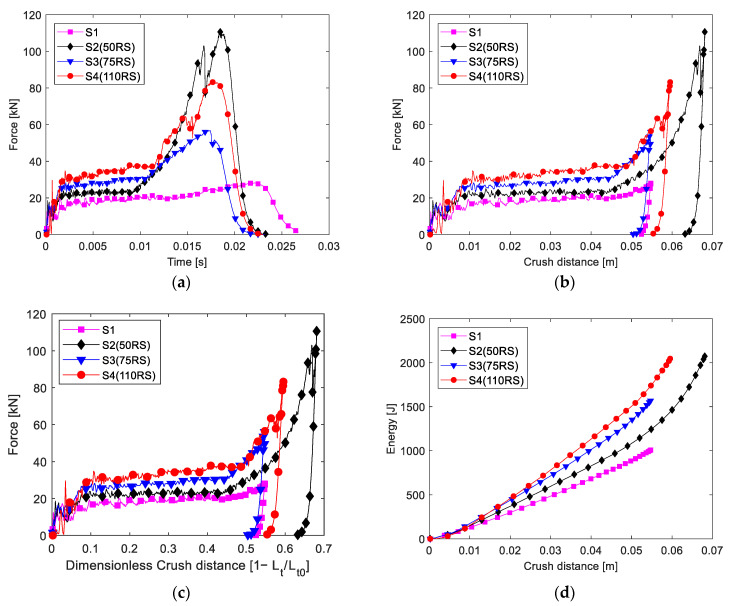
Experimental results of the 2.0 mm-thick samples: (**a**) load and time, (**b**) load and distance, (**c**) load and dimensionless crush distance, and (**d**) energy absorbed and crush distance.

**Figure 8:**
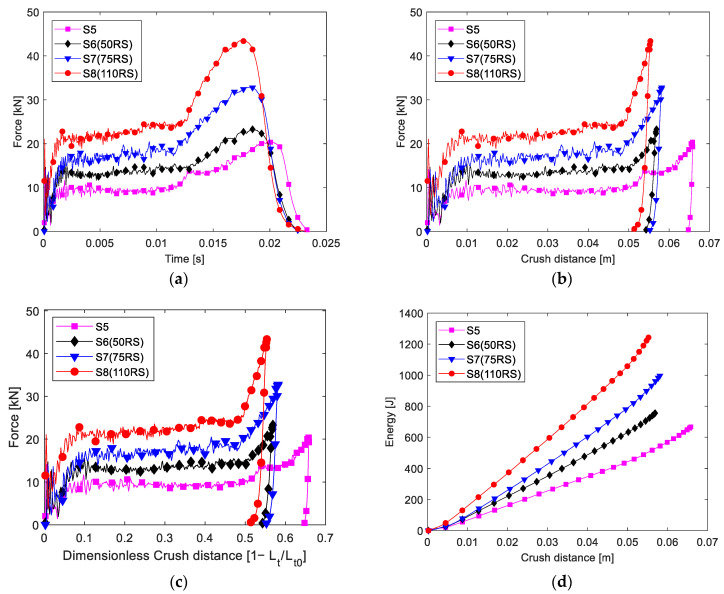
Experimental results of the 1.5 mm-thick samples: (**a**) load and time, (**b**) load and distance, (**c**) load and dimensionless crush distance, and (**d**) energy absorbed and crush distance.

The time histories of the impact load recorded in the impact test (Figure 7a and Figure 8a) were used to calculate the crush distance, and then, the absorbed energy was calculated. As the impact velocity and impact energy were not the same in different tests, the time histories of the impact load were not synchronous. Further, the crush load was plotted with the crush distance and dimensionless load (Figure 7b,c and Figure 8b,c). Evidently, two main stages were observed after the initial failure. First was the stable stage corresponding to when the destructed tube was pushed into the tube hollow; essentially, the folded composite frond squeezed the foam, which, in turn, blocked the movement of the composite tube and increased the blocking force of the absorber. Second is the load-increasing stage after the crush distance was greater than half of the tube; essentially, the fronds reached one pressing cap and began to be compressed further.

The initial peak load of the absorber filled with the 70 RS and 110 RS foams was lower than that of the MCF, and the CFE was greater than 1 according to Equation (6). Therefore, in this study, the MICF was determined as the maximum load in the steady stage. The MICF, MCF, CFE, and SEA values in the stable stage of each type of absorber are listed in Table 3. Evidently, the CFE of the foam-filled absorber was greater than 0.86.

It should be mentioned that the maximum force is not discussed, which is located in the last stage in the curve dominated by the reaction of the compacted debris at the final crush distance. Some changes of the impact energy or absorber parameters affected the final crush distance and the maximum force, so the maximum force in the last stage was an incredible factor for the presented tests.

**Figure 9:**
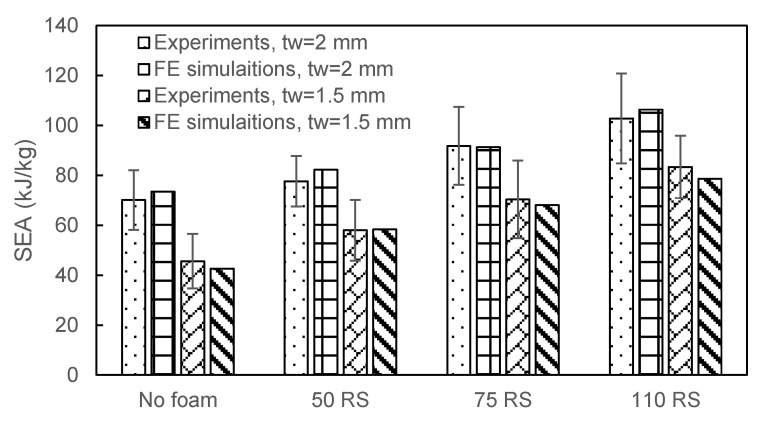
SEA comparison of absorber with different wall thicknesses and foams.

The SEA values of different specimens were also compared, as shown in Figure 9. The increment in the SEA from foam ranged from 10 to 46% for the 2 mm-thick tube and from 27 to 85% for the 1.5 mm-thick tube. The 2.0 mm-thick tube without foam had a higher SEA. Note that the higher the elastic modulus of the foam, the higher the energy absorption achieved was. Herein, the best SEA was achieved by the 2.0 mm-thick tube filled with the 110 RS foam (SEA, 103 kJ/kg), 47% higher than that of the 2.0 mm-thick tube without foam.

The energy absorbed was plotted against the crush distance, as shown in Figure 7d and Figure 8d, where the slope of the curve reflects the SEA; evidently, the slope increased after the dimensionless crush distance was greater than 0.5.

### 4.2. Simulation Results

**Figure 10:**
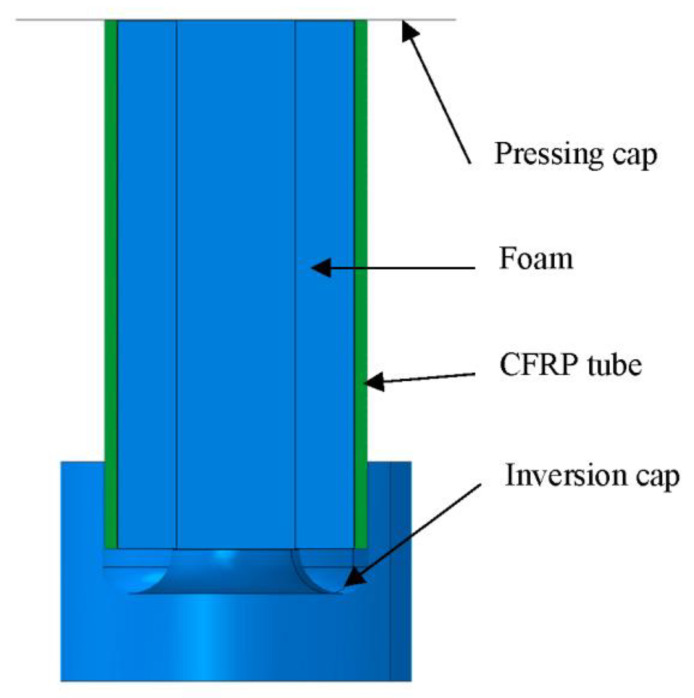
Components of FE model.

Numerical models were established using ABAQUS; the parts of the FE model are shown in Figure 10. The pressing and inversion caps were modelled using a rigid material. The length of the composite tube was 60 mm in the FE model to reduce the computational time. General contact was applied to the model with a friction factor of 0.27 between the composites and 0.12 between the composites and inversion cap. The inversion cap was fixed at the reference point. An initial velocity of 2.5 m/s was applied to the pressing cap.

**Figure 11:**
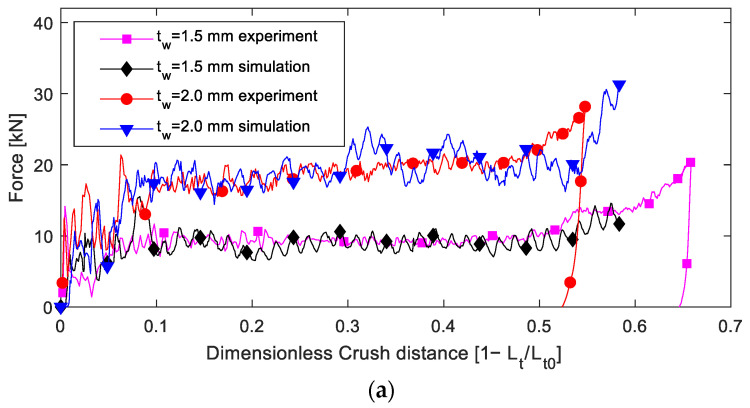

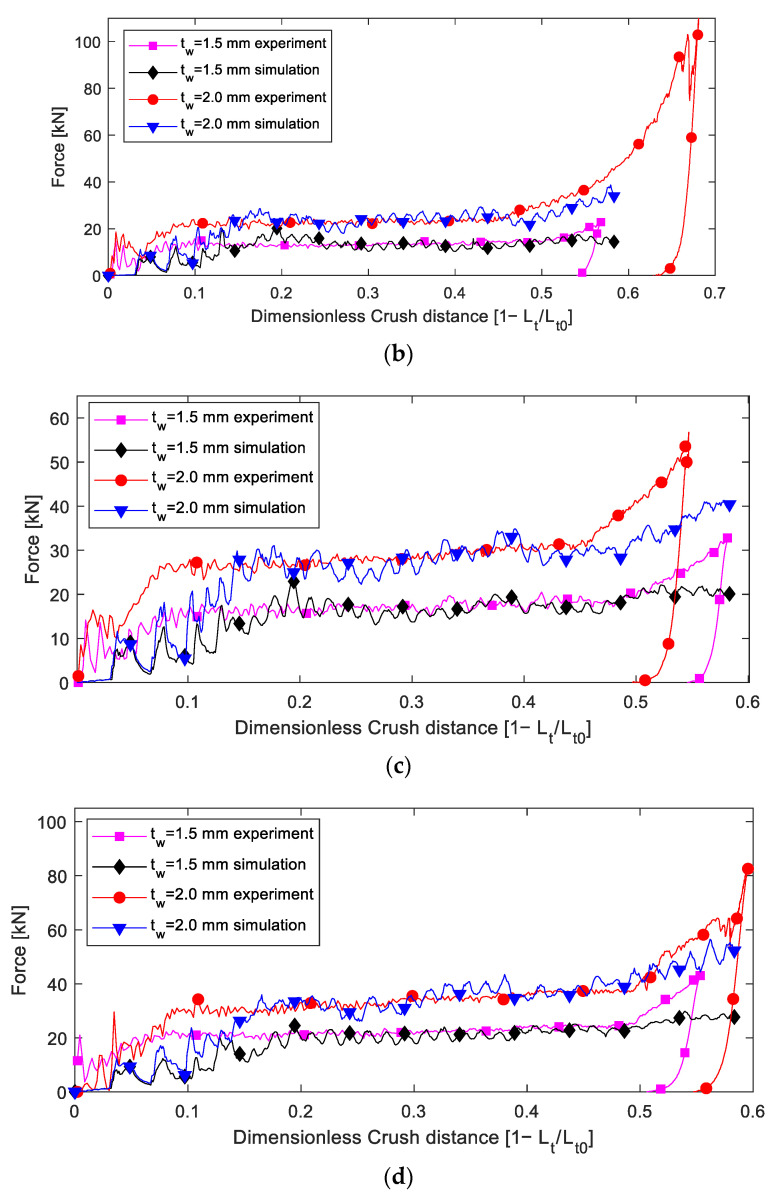
Comparison of impact load from the experimentation and simulation: (**a**) without foam, (**b**) with 50 RS foam, (**c**) with 75 RS foam, and (**d**) with 110 RS foam.

As the tube length in the simulation was different from that in the experiment, the load curves were plotted with the dimensionless crush distance and for comparison, as shown in Figure 11. Evidently, the simulation curve agreed well with the experimental results in the stable stage. It should be noted that the simulated load in the initial dimensionless crush distance (within 0.15) was lower than the experimental one, due to the tube length (60 mm) in the simulation being different from that of the experiment (100 mm). The behavior of the sample in the load-increasing stage was complicated to simulate using the presented FE model; therefore, the simulation was stopped when the crush distance exceeded half of the tube length. The SEA of the stable stage from the simulation was compared with that from the experiment, as listed in Table 3, where the maximum error was less than 7%.

To further investigate the failure of the composite tube, the damage morphologies of Sample S2 at different crush distances obtained from the quasi-static compressive test are given in Table 7, and the cross-section of Sample S2 after the impact test is also given. Correspondingly, the damage factors of the four failure modes shown in Table 4 are also given for each crush stage, respectively.

When the crush distance was equal to 3 mm, the tube end began to be compressed in the radial direction under the effect of the inverted surface. There were fiber breakages and matrix cracks along the periphery. Delaminations also occurred at the tip. According to the simulation results, all damage modes began to emerge, except the fiber tensile failure.

The plies were compressed further and began to intersect when the crush distance was equal to 4.5 mm, and fiber tensile failure occurred due to fiber bending. Other types of damage grew, especially the matrix crushing due to in-plane compression.

The tip of composite tube was completely inverted after the crush distance became greater than 10 mm, while most plies were bent, then fiber tensile failure was presented in the simulation results. Other failures developed, in which the quantity of fiber compressive failure was relatively less due to the buckling of the thin ply.

From the cross-section of the sample after the impact test, the composite wall was destructed by the internal inversion cap; the fronds were inverted and compacted; the foam was also compressed. The collapse and buckling of the composite fronds in the simulation were similar to those in the experimentation. The damage factors showed that the plies were destructed seriously, which is beneficial for energy absorption.

## 5. Conclusions

The crush performance under the internal inversion cap of composite tubes with 1.5 mm and 2 mm thicknesses filled with different PMI foams was investigated. The experimental results revealed that the 2 mm-thick tube had a higher SEA than the 1.5 mm-thick tube. Evidently, the higher the elastic modulus of the filled foam, the higher the SEA achieved was. The best SEA was achieved by the 2.0 mm-thick tube filled with the 110 RS foam, with a value of 103 kJ/kg, which was 47% higher than that of the 2.0 mm-thick tube without foam.

A numerical simulation of the collapse of the foam-filled tubes based on ABAQUS/Explicit was conducted. The material behaviors of the composite tube were defined using the VUMAT user subroutine. The parameters of the foam model were determined through experimentation on the foam. The improved Chang–Lessard failure criteria were used to judge the damage initiation; subsequently, the stiffness of the element was reduced through the VUMAT user subroutine. The initial failure and stable stage of the crushing were simulated. The results revealed that the mean crush force from the simulation was in good agreement with the experimental data with errors of the SEA less than 7%, thus indicating that the method is appropriate for the crush simulation of the proposed shock absorber. The development of six damage modes to the plies were analyzed, five of which were developed thoroughly, except the fiber compressive failure, showing a good energy absorption mechanism under the action of the internal inversion cap.

The shock absorber proposed in this study has a long stroke with a steady reaction load and a high SEA, and therefore, it has good prospects for crashworthiness engineering structures. For cars, it can be used in the crash box behind the front bumper. A shock absorber with a similar configuration as that in this study, but not filled with foam, was utilized in the legged landing gear of a drone [26]. According to the existing research, the shock absorber could be used in the subfloor stanchion of passenger airplanes [9,25]. Further studies will focus on the application to engineering structures and the improvement of the SEA through ply sequence optimization.

## Figures and Tables

**Table 1:** Mechanical properties of and symbols for carbon-fiber/epoxy prepreg unidirectional tape.

Properties	Value
Density	1529 kg/m^3^
Young’s modulus *E*_1_	115 GPa
Young’s modulus *E*_2_ = *E*_3_	8 GPa
$\mathrm{Poisson}’s\;\mathrm{ratio}\;\nu_{12}$	0.3
$\mathrm{Poisson}’s\;\mathrm{ratio}\;\nu_{13}$	0.3
$\mathrm{Poisson}’s\;\mathrm{ratio}\;\nu_{23}$	0.35
Shear modulus *G*_12_ = *G*_13_	4.9 GPa
Shear modulus *G*_23_	3 GPa
Tensile fracture toughness $G_{1C}^{t}$	133 N/mm
Compressive fracture toughness $G_{1C}^{c}$	40 N/mm
Tensile fracture toughness $G_{2C}^{t}$ $=G_{3C}^{t}$	0.6 N/mm
Compressive fracture toughness $G_{2C}^{c}=G_{3C}^{c}$	2.1 N/mm
Tensile strength *X*_t_	1600 MPa
Compressive strength *X*_c_	1000 MPa
Tensile strength *Y*_t_	40 MPa
Compressive strength *Y*_c_	135 MPa
Tensile strength *Z*_t_	40 MPa
Compressive strength *Z*_c_	135 MPa
Shear strength *S*_12_ = *S*_13_ = *S*_23_	89 MPa

**Table 2:** Mechanical properties of PMI foam.

Type	Density (kg/m^3^)	Compressive Strength (MPa)	Tensile Strength (MPa)	Young’s Modulus (MPa)	Fracture Elongation (%)	Shear Strength (MPa)	Shear Modulus (MPa)
50 RS	50	0.85	1.68	83	2.6	0.85	30
75 RS	70	1.70	2.30	108	2.8	1.25	48
110 RS	110	3.60	3.70	197	2.8	2.38	80

**Table 3:** Parameters of tested specimens and characteristics of energy absorption.

Type of Absorber	*t*_w_ (mm)	Foam	MICF (kN)	MCF in Stage 1 (kN)	CFE	SEA (kJ/kg)	SEA from Simulation (kJ/kg)	Error of SEA Simulation (%)
S1	2	None	21.5	18.8	0.87	70.1	73.5	4.59
S2	2	50 RS	25.9	22.9	0.88	77.6	82.2	5.68
S3	2	75 RS	31.5	28.2	0.90	91.8	91.3	−0.56
S4	2	110 RS	38.3	33.5	0.87	102.8	106.3	3.32
S5	1.5	None	14.2	9.4	0.66	45.6	42.7	−6.81
S6	1.5	50 RS	15.8	13.5	0.866	58.0	58.3	0.51
S7	1.5	75 RS	22.2	17.4	0.78	70.4	68.1	−3.38
S8	1.5	110 RS	22.8	22.2	0.98	83.4	78.7	−5.97

**Table 4:** Failure criteria for the unidirectional ply.

Failure Modes	Damage Factors
$\mathrm{Fiber}\;\mathrm{tensile}\;\mathrm{failure}\;\mathrm{criterion}\;(\sigma_{1}\geq 0$)	$F_{\mathrm{ft}}=\sqrt{{\left(\frac{\sigma_{1}}{X_{T}}\right)}^{2}+{\left(\frac{\tau_{12}}{S_{12}}\right)}^{2}+{\left(\frac{\tau_{13}}{S_{13}}\right)}^{2}}\geq 1$
$\mathrm{Fiber}\;\mathrm{compressive}\;\mathrm{failure}\;(\sigma_{1}<0$)	$F_{\mathrm{fc}}=\sqrt{{\left(\frac{\sigma_{1}}{X_{C}}\right)}^{2}}\geq 1$
$\mathrm{Matrix}\;\mathrm{tensile}\;\mathrm{failure}\;\mathrm{in}\;2\;\mathrm{directions}\;(\sigma_{2}\geq 0$)	$F_{\mathrm{mt}}=\sqrt{{\left(\frac{\sigma_{2}}{Y_{T}}\right)}^{2}+{\left(\frac{\tau_{12}}{S_{12}}\right)}^{2}+{\left(\frac{\tau_{23}}{S_{23}}\right)}^{2}}\geq 1$
$\mathrm{Matrix}\;\mathrm{crushing}\;\mathrm{failure}\;\mathrm{criterion}\;\mathrm{for}\;\mathrm{in}\text{-}\mathrm{plane}\;\mathrm{compression}\;(\sigma_{2}<0$)	$F_{\mathrm{mc}}=\sqrt{{\left(\frac{\sigma_{2}}{Y_{C}}\right)}^{2}+{\left(\frac{\tau_{12}}{S_{12}}\right)}^{2}+{\left(\frac{\tau_{23}}{S_{23}}\right)}^{2}}\geq 1$
$\mathrm{Matrix}\;\mathrm{crushing}\;\mathrm{failure}\;\mathrm{criterion}\;\mathrm{for}\;\mathrm{out}\text{-}\mathrm{of}\text{-}\mathrm{plane}\;\mathrm{tension}\;(\sigma_{3}\geq 0$)	$F_{\mathrm{dt}}=\sqrt{{\left(\frac{\sigma_{3}}{Z_{T}}\right)}^{2}+{\left(\frac{\tau_{13}}{S_{13}}\right)}^{2}+{\left(\frac{\tau_{23}}{S_{23}}\right)}^{2}}\geq 1$
$\mathrm{Matrix}\;\mathrm{crushing}\;\mathrm{failure}\;\mathrm{criterion}\;\mathrm{for}\;\mathrm{out}\text{-}\mathrm{of}\text{-}\mathrm{plane}\;\mathrm{compression}\;(\sigma_{3}<0$)	$F_{\mathrm{dc}}=\sqrt{{\left(\frac{\sigma_{3}}{Z_{C}}\right)}^{2}+{\left(\frac{\tau_{13}}{S_{13}}\right)}^{2}+{\left(\frac{\tau_{23}}{S_{23}}\right)}^{2}}\geq 1$
$\sigma_{1},\sigma_{2},\;\sigma_{3}$—normal stress; $\tau_{12},\tau_{13},\;\tau_{23}$—shearing stress; $F_{k}$ (*k* = ft, fc, mt, mc, dt, dc)—damage factor.	

**Table 5:** Properties of cohesive model.

Properties	Values
$E_{nn}$	3000 MPa
$E_{ss}$ $=E_{tt}$	1154 MPa
$t_{n}^{0}$	38 MPa
$t_{s}^{0}=t_{t}^{0}$	55 MPa
$G_{n}^{C}$	0.533 N/mm
$G_{s}^{C}$ $=G_{t}^{C}$	1.1 N/mm

**Table 6:** Parameters of foam for FE simulation.

Type of Foam	50 RS	75 RS	110 RS
$\mu_{1}$	−24.59	−39.53	−71.04
$\mu_{2}$	45.99	80.15	146.54
$\mu_{3}$	−1.17	−2.18	−4.00
$\alpha_{1}$	16.74	15.40	15.26
$\alpha_{2}$	25.00	25.00	25.00
$\alpha_{3}$	−1.25	−1.24	−1.24
$\nu_{1}$	0.045	0.045	0.045
$\nu_{2}$	0.045	0.045	0.045
$\nu_{3}$	0.045	0.045	0.045

**Table 7:** Morphologies of the specimen damage at different crush distances and the corresponding damage factors of FE simulation.

Crush Stage	*δ* = 3 mm	*δ* = 4.5 mm	*δ* = 10 mm	Cross-Section of Impacted Test Sample
Damage morphology	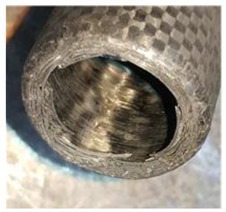	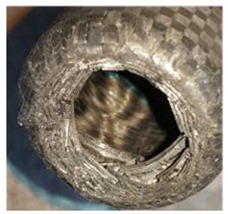	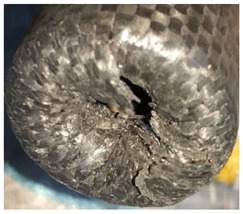	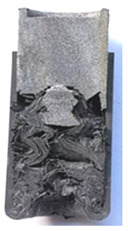
$F_{ft}$	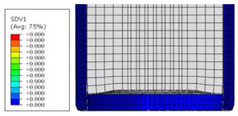	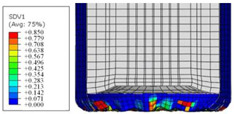	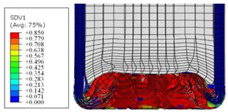	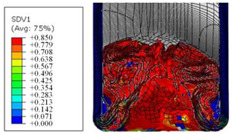
$F_{fc}$	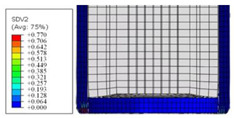	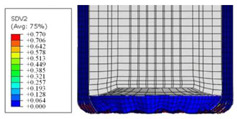	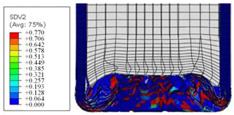	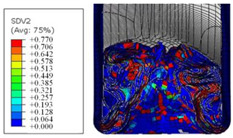
$F_{mt}$	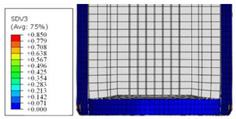	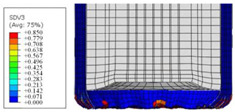	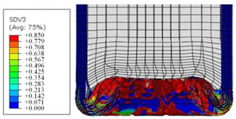	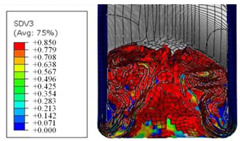
$F_{mc}$	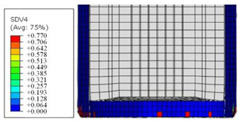	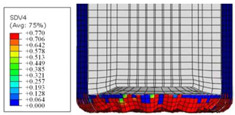	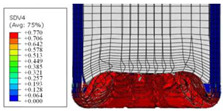	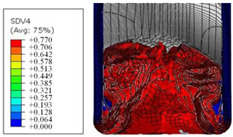
$F_{dt}$	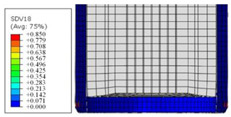	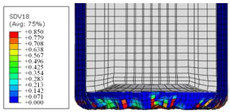	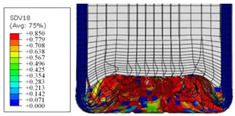	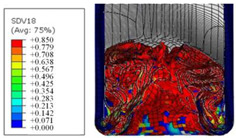
$F_{dc}$	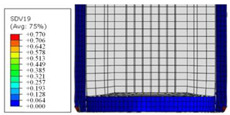	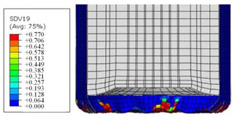	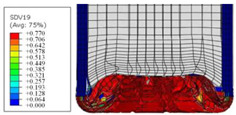	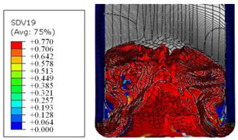

## Data Availability

The data presented in this study are available upon request from the corresponding author.
